# Flotillin membrane domains in cancer

**DOI:** 10.1007/s10555-020-09873-y

**Published:** 2020-04-15

**Authors:** Cécile Gauthier-Rouvière, Stéphane Bodin, Franck Comunale, Damien Planchon

**Affiliations:** CRBM, University of Montpellier, CNRS, Montpellier, France

**Keywords:** Flotillins, Cancer, Vesicular trafficking, Signaling

## Abstract

Flotillins 1 and 2 are two ubiquitous, highly conserved homologous proteins that assemble to form heterotetramers at the cytoplasmic face of the plasma membrane in cholesterol- and sphingolipid-enriched domains. Flotillin heterotetramers can assemble into large oligomers to form molecular scaffolds that regulate the clustering of at the plasma membrane and activity of several receptors. Moreover, flotillins are upregulated in many invasive carcinomas and also in sarcoma, and this is associated with poor prognosis and metastasis formation. When upregulated, flotillins promote plasma membrane invagination and induce an endocytic pathway that allows the targeting of cargo proteins in the late endosomal compartment in which flotillins accumulate. These late endosomes are not degradative, and participate in the recycling and secretion of protein cargos. The cargos of this Upregulated Flotillin–Induced Trafficking (UFIT) pathway include molecules involved in signaling, adhesion, and extracellular matrix remodeling, thus favoring the acquisition of an invasive cellular behavior leading to metastasis formation. Thus, flotillin presence from the plasma membrane to the late endosomal compartment influences the activity, and even modifies the trafficking and fate of key protein cargos, favoring the development of diseases, for instance tumors. This review summarizes the current knowledge on flotillins and their role in cancer development focusing on their function in cellular membrane remodeling and vesicular trafficking regulation.

## Membrane domains

Membrane micro-domains, also called “lipid rafts,” are highly ordered membrane subdomains enriched in cholesterol, sphingolipids, and gangliosides and with roles in membrane organization, lateral movement and trafficking of molecules, and signal transduction. Several signal transduction processes involved in cell adhesion and migration and in the formation of sorting platforms for targeted protein trafficking are dependent on this precise membrane organization. The first evidence of the presence of heterogeneous domains in the cell membrane came from biochemical studies based on their insolubility in non-ionic detergent at low temperature and their isolation by flotation in sucrose gradient, hence their name detergent-resistant membrane (DRM) domains. Although their *in vivo* presence was unclear at that time, new technologies, particularly super-resolution imaging and spectroscopy techniques and lipid analysis, provided data to better define these “lipid rafts” as heterogeneous and highly dynamic domains that range from 10 to 300 nm in size. Lipid-lipid and protein-lipid interactions and clustering allow the formation of these heterogeneous membrane domains that dynamically tune the bioactivity of cell membranes [1, 2].

These membrane domains have crucial roles in the regulation of adhesion, cell signaling pathways, protein sorting, and trafficking, all key processes in cancer development. Thus, alterations in membrane domain homeostasis (at the lipid and/or protein level) may directly promote changes in these processes, leading to tumorigenesis.

Flotillin 1 and flotillin 2, when overexpressed, might perturb membrane domain homeostasis during tumorigenesis. In the last years, our vision of flotillin role has drastically evolved. Several convincing studies in cellular models have demonstrated the role of flotillins in the establishment of protein complexes at the plasma membrane and in endocytosis; however, flotillin-deficient mice are viable and fertile [3, 4]. Some insights came from the observation that flotillins are upregulated in major human pathologies, particularly in many tumors where their overexpression correlates with poor prognosis (Table 1). Consistently, flotillin co-upregulation in various cellular models promotes cell invasion and metastasis [5–11], and flotillin 2 knockout mice allowed demonstrating the crucial role of flotillins in metastasis formation [12]. This led to the notion of gain-of-function effect of flotillins when they are upregulated. Indeed, upregulated flotillins promote plasma membrane invagination and endocytosis towards late endosomes, thus modifying the trafficking of different cargos. Therefore, the Upregulated Flotillin–Induced Trafficking (UFIT) pathway changes cell fate and promotes tumorigenesis.

**Table 1:** Flotillin 1 and 2 expression levels in cancers

Cancer type	Flotillin detection	Observed defect	Mechanisms	Link with oncogenic pathways	Source
From epithelial origin (carcinoma)					
Breast	FLOT2, cDNA microarrays (42 individuals, 3 normal samples)	Upregulation	Amplification (of *ERBB2* locus)	n.d.	[13]
	FLOT2, IHC (194 individuals, no normal sample)	Upregulation, poor prognosis	n.d.	FLOT1 KD decreases ERBB2 level	[14]
	FLOT2, IHC (117 individuals, no normal sample) + 1 normal and 8 breast cell lines	Upregulation, poor prognosis	n.d.	n.d.	[15]
	FLOT1, IHC (78 individuals, 40 normal samples) + 2 normal and 7 tumoral cell lines	Upregulation, poor prognosis	Mir-124 target	n.d.	[16]
	FLOT2 (17 individuals + 50 cell lines, no normal sample)	Upregulation, metastasis formation	Amplification with ERBB2	Flot2 KO decreased metastasis formation without effect on primary tumor formation*	[12]
	FLOT1, IHC (289 individuals, no normal sample)	Upregulation, poor prognosis	Correlation with H-ras expression	FLOT1 KD decreased activated H-Ras, AKT, Rac1, p38	[7]
	FLOT1&2, RT-qPCR (527, no normal sample)	Upregulation, poor prognosis	n.d.	FLOT1 KD decreased MT1-MMP-mediated matrix degradation. FLOT2 expression level is correlated with tumor cell invasion**	[9]
Cervical	FLOT2, IHC (115 individuals, 10 normal samples)	Upregulation, poor prognosis	n.d.	n.d.	[17]
	FLOT2, IHC (115 individuals, 5 normal samples + 1 normal and 5 tumoral cell lines)	Upregulation, poor prognosis	n.d.	n.d.	[18]
Colorectal	FLOT1, IHC (81 individuals: tumors and adjacent tissues)	Upregulation, poor prognosis	n.d.	n.d.	[19]
	FLOT2, IHC (180 individuals + 1 normal and 5 tumoral cell lines)	Upregulation, poor prognosis	n.d.	n.d.	[20]
Esophageal squamous cell carcinoma	FLOT1, IHC (432 individuals, 8 normal samples + 2 normal and 11 tumoral cell lines)	Upregulation, poor prognosis	n.d.	TNFα/NF-kB activation	[21]
Gastric	FLOT2, IHC (282 individuals, no normal sample)	Upregulation, poor prognosis	Amplification with ERBB2	n.d.	[22]
Head and neck	FLOT2, RT-qPCR (81 individuals, no normal sample)	FLOT2 belongs to a four-gene signature predictive of metastasis	n.d.	n.d.	[23]
Hepatocellular carcinoma	FLOT1, IHC (196 individuals, 10 normal samples + 1 normal and 14 tumoral cell lines)	Upregulation, poor prognosis	n.d.	n.d.	[24]
	FLOT2, IHC (187 individuals, 2 normal samples + 1 normal and 7 tumoral cell lines)	Upregulation, poor prognosis	n.d.	FLOT2 expression is correlated with MEK/Raf/ERK activation. FLOT2 expression level is correlated with tumor growth and metastasis formation**	[11]
Lung	FLOT1, IHC (108 individuals, 5 control samples)	Upregulation, poor prognosis	n.d.	n.d.	[25]
Non-small-cell lung cancer (NSCLC)	FLOT2, RT-qPCR (24 individuals: tumors and adjacent tissues), IHC (90 individuals, 1 normal and 7 tumoral cell lines)	Upregulation, poor prognosis	n.d.	n.d.	[26]
	FLOT2, IHC (352 individuals, 59 control samples)	Upregulation, poor prognosis	n.d.	n.d.	[27]
Melanoma	FLOT2, IHC (182 individuals + 11 tumoral cell lines)	Upregulation	n.d.	FLOT2 expression level is correlated with metastasis formation but not with tumor growth**	[6]
	FLOT2, IHC (38 individuals, no normal sample)	Upregulation, poor prognosis, lymph node metastases	n.d.	n.d.	[28]
Nasopharyngeal	FLOT2, IHC (181 individuals, tumors and adjacent tissues)	Upregulation, poor prognosis	n.d.	FLOT2 participates in TGFβ-induced EMT	[29]
	FLOT1, IHC (169 individuals + 1 normal and 6 tumoral cell lines)	Upregulation, poor prognosis, lymph node metastases	n.d.	FLOT2 activates the TGFβ pathway	[5]
	FLOT2, IHC (132 individuals, 38 control samples)	Upregulation, poor prognosis	n.d.	FLOT2 activates NF-kB and PI3K/AKT	[8]
Oral squamous cell carcinoma	FLOT1, IHC (181 individuals, no normal sample)	Upregulation, poor prognosis	n.d.	n.d.	[30]
	FLOT2, IHC (78 individuals, 27 normal samples)	Upregulation, poor prognosis	n.d.	n.d.	[31]
Renal	FLOT2, IHC (106 individuals)	Upregulation, poor prognosis	n.d.	n.d.	[32]
	FLOT1, RT-qPCR (182 individuals: tumors and adjacent tissues)	Upregulation, poor prognosis	n.d.	n.d.	[33]
From non-epithelial origin: mesenchymal origin (sarcoma), neuronal origin					
Liposarcoma	FLOT1, RT-qPCR (15 individuals, no normal sample)	Downregulation	n.d.	n.d.	[34]
Other sarcoma (synovial, histiocytoma, schwannoma)	FLOT1, RT-qPCR (18 individuals, no normal sample)	Upregulation	n.d.	n.d.	[34]
Glioma	FLOT2, IHC (56 individuals: tumors and adjacent tissues + 1 normal and 5 tumoral cell lines)	Upregulation, poor prognosis	miR-449 target	n.d.	[35]
Pediatric tumors					
Neuroblastoma	FLOT1, RT-qPCR (88 individuals), WB (45 individuals)	Downregulation, poor prognosis	n.d.	FLOT1 controls ALK distribution and activity	[10]
Rhabdomyosarcoma	FLOT1&2, RT-qPCR (81 individuals, 1 normal sample)	Upregulation, poor prognosis	n.d.	n.d.	[9]

*FLOT1* flotillin 1, *FLOT2* flotillin 2, *IHC* immunohistochemistry, *WB* Western blotting, *n.d.* not determined

Number between brackets indicates the number of analyzed samples

*Data obtained using mouse models

**Xenografts (mice or zebrafish)

## Flotillins

Flotillins were concomitantly discovered by two different groups as proteins upregulated in mammalian retinal ganglion cells upon optic nerve injury (hence their name Reggie) [36] and as markers of lipid raft domains in plasma membrane extracts from mouse lung tissue (hence their name flotillins) [37].

Flotillin-like proteins are found in several organisms such as bacteria, the social amoeba *Dictyostelium discoideum*, fungi, and plants, but are absent in *Caenorhabditis elegans* and budding yeast. In all these organisms, flotillins were found in DRM, when tested. In bacteria, flotillins are present in DRM domains and can be visualized as discrete membrane regions by fluorescent microscopy. Bacterial flotillins regulate membrane fluidity, act as scaffold proteins for efficient protein complex assembly, and participate in the formation of structures that promote membrane fusion and invagination during cell division and sporulation [38]. In *Dictyostelium discoideum*, three flotillin-like proteins, VacA, VacB, and VacC, are associated with membrane domains and participate in particle uptake, plasma membrane recycling, and phagolysosome biogenesis [39]. In various plant genomes, flotillin homologs were identified. For instance, in *Arabidopsis thaliana*, there are three homologs that are detected in plasma membrane DRM [40].

**Fig. 1:**
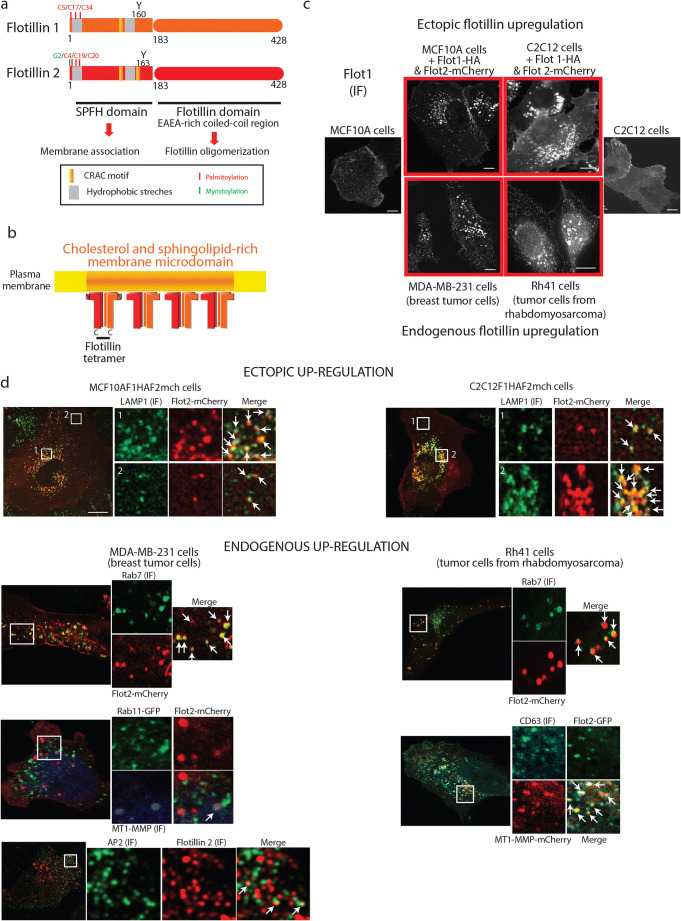
Flotillin 1 and 2 structures and localization. **a** Representation of the main domains and residues in the flotillin 1 and 2 sequences. SPFH (stomatin, prohibitin, flotillin, HflK/C) domain (from amino acids 5 to 183 and from 7 to 183 in flotillins 1 and 2, respectively) is also known as the Prohibitin homology domain (PHD) [41]. This domain mediates the association with cholesterol-rich membrane micro-domains. The different palmitoylation sites are shown. Flotillin 2 is also myristoylated on G2 of the unstructured motif that is upstream the SPFH domain and is required for its membrane association [47]. Other motifs could be involved in the membrane association of flotillins: the hydrophobic stretches and CRAC (cholesterol recognition amino acid consensus) motifs [110, 111]. Flotillin oligomerization is mostly dependent on coiled-coil regions in the C-terminal flotillin domains. Phosphorylation of the tyrosine residues Y160 and Y163 (flotillin 1 and 2, respectively) participates in flotillin hetero-oligomerization [46, 58]. **b** Schematic view of flotillin oligomerization and the formation of flotillin platforms in cholesterol- and sphingolipid-rich membrane domains. **c** Flotillin intracellular distribution is dependent on their expression level. At the physiological expression level, flotillins are located at the plasma membrane and in intracellular vesicles. When they are upregulated (either endogenous upregulation in invasive tumor cells or upon ectopic overexpression in normal cells), they mainly accumulate in intracellular vesicles. The figure shows confocal micrographs of cells of epithelial (MCF10A and MDA-MB-231) and mesenchymal origin (C2C12 and Rh41) stained with an anti-flotillin 1 antibody as described [9]. Bar, 10 μm. **d** When upregulated (either endogenous overexpression in invasive MDA-MB-231 and Rh4 tumor cells or ectopic overexpression in normal MCF10AF1F2 and C2C12F1F2 cells), flotillins accumulate in perinuclear and peripheral vesicles that express late endosomal markers (LAMP-1, CD63, or Rab7). Few flotillin vesicles co-localize with Rab11, a marker of recycling endosomes, and with Rab4, Rab8, CD9, and CD91 (for the full description, see 7). Some flotillin vesicles co-localize with the early endocytic markers EEA1 and Rab5, and correspond to flotillin-rich endocytic vesicles [9]. MT1-MMP, a protein cargo of the UFIT pathway, is present in the flotillin-positive late endosomes (shown in Rh4 cells and not shown in MDA-MB-231 cells). Bar, 10 μm. Methods: Cells were fixed in 3.2% paraformaldehyde (in phosphate-buffered saline, PBS) for 15 min, followed by a 2-min permeabilization with 0.1% Triton X-100 (in PBS) and saturation with 2% BSA (in PBS). For CD63 detection, cells were permeabilized using 0.1% saponin. Cells were incubated with primary and secondary antibodies in PBS containing 2% BSA. Confocal images were acquired using a Confocal Leica SP5-SMD microscope and a LEICA 63x/1.4 oil HCX PL APO CS objective controlled using the Leica LAS AF software. Primary antibodies used: Mouse antibodies against flotillin 1 (1:1000, 610820, BD Biosciences), flotillin 2 (1:100, 610383, BD Biosciences), Rab7 (1:400, 50533, Abcam), LAMP1 (1:500, 555798, BD Biosciences), AP2 (1:100, 610501, BD Bioscience), and CD63 (1:50, clone R5G2, MBL). Alexa 350 488, 546 dye-conjugated secondary antibodies were from Thermo Scientific

Flotillins are composed of two domains (Fig. 1a): the N-terminal SPFH (stomatin, prohibitin, flotillin, HflK/C) domain associated with the inner leaflet of cell membranes and the C-terminal flotillin domain that is found only in flotillins 1 and 2 and is responsible for flotillin oligomerization [41, 42]. Although flotillins can form homo-tetramers, flotillin hetero-oligomers (composed of two flotillin 1 and two flotillin 2) are the predominant form. Flotillin hetero-oligomers assemble into large oligomers that form flotillin platforms at the membrane [42] (Fig. 1b). Micro-domains formed by oligomerized flotillins are distinct from those scaffolded by caveolins. Flotillins are interdependent regarding their functions and stability. Indeed, the decreased expression of one flotillin results in the reduced expression also of the other [4, 43–46].

The SPFH domain allows the association of flotillins with cholesterol-rich membrane domains *via* interaction with the hydrophobic amino acid stretches or with putative CRAC (cholesterol recognition amino acid consensus) motifs and through post-translational modifications (palmitoylation for flotillin 1, and both palmitoylation and myristoylation for flotillin 2) [47]. The SPFH domain of flotillin 2 can bind to actin, and this interaction stabilizes flotillin domains. Indeed, actin depolymerization increases their mobility [48–50]. Flotillins are mainly localized in membrane compartments, such as the plasma membrane, the endoplasmic reticulum (ER), the Golgi apparatus, and a variety of late endosomal compartments, characterized as endolysosomes, phagosomes, or multi-vesicular endosomes, depending on the cellular model analyzed [9, 45, 51, 52]. Whether flotillins are transported from the ER directly to the plasma membrane or whether they pass through intermediate compartments is unknown. The secretion pathway of flotillin 1 is apparently unconventional (i.e., Golgi-independent) [41]. Flotillin 1 palmitoylation at Cys34 is required for its exit from the ER and its localization at the plasma membrane [41, 53, 54]. Some data obtained in HeLa cells incubated with brefeldin A, to inhibit transport from the Golgi apparatus, suggest that flotillin 2 could use the conventional secretory pathway [45]. The presence of flotillins 1 and 2 in the Golgi apparatus was also confirmed in experiments where proteins present in Golgi-derived detergent-insoluble complexes were sequenced and by immuno-electron microscopy showing flotillin 2 in Golgi vesicles but not in Golgi stacks [41, 45, 55]. Moreover, the expression of truncated flotillins 1 and 2 bearing only their SPFH domains results in their retention in the Golgi, but this is not observed with the full-length proteins. Post-translational modifications could also influence flotillin distribution. Interestingly, non-palmitoylable flotillin 1 appears to be more prone to sumoylation, a post-translational modification that promotes flotillin 1 nuclear translocation [56]. The expression level of flotillins also has a strong impact on their cellular distribution (Fig. 1c). At low expression levels, flotillins mainly reside at the inner leaflet of the plasma membrane, whereas they accumulate in late endosomes when upregulated, for example in tumor cells [9]. Flotillin redistribution from the plasma membrane to late endosomes is induced also by incubation with EGF [57, 58]. In HeLa cells, this effect involves dynamin [59]. It has been suggested that Fyn-dependent phosphorylation of flotillin 1 (Y160) and flotillin 2 (Y163) induces their endocytosis. Moreover, these phosphorylatable residues appear to be important for flotillin hetero-oligomerization and their subsequent endocytosis [46]. As flotillin upregulation favors their oligomerization, it will be interesting to analyze whether this affects also Fyn activation. Recently, it was shown that the use of ultrasound in combination with microbubbles, a strategy for targeted intracellular delivery of molecules, elicits a signaling pathway involving Fyn and the palmitoyl transferase DHHC5, which in turn triggers an increase in flotillin internalization [60].

## Flotillin role in tumorigenesis

Flotillins are upregulated in a subset of all carcinomas and also in sarcomas, and this is associated with poor patient prognosis (see Table 1 for an up-to-date census).

In the last years, some studies have started to identify how flotillins are upregulated in cancer. Not surprisingly, flotillin expression is regulated through mechanisms that are perturbed in tumor cells. Indeed, flotillin upregulation might be caused by microRNA downregulation in tumors (Table 2). For instance, microRNA-802 is downregulated in breast, pancreatic, and prostate cancers. In prostate cancer, this microRNA controls the expression of genes associated with epithelial to mesenchymal transition (EMT) and directly targets flotillin 2 [61]. Flotillin 1 is a microRNA-124 target in breast tumors [16], and flotillin 2 is a microRNA-485-5p target in non-small-cell lung cancer [62]. In gastric cancer, microRNA-485-5p targets flotillin 1 and microRNA-449a flotillin 2 [63, 64]. Gene amplification also might increase flotillin expression level. Specifically, in breast and gastric cancers, flotillin 2 is co-amplified with *ERBB2* (these two genes are close on chromosome 17) [13, 22]. At the transcriptional level, flotillin 2 is a direct target of TAp73β and TAp63γ, two p53 family members [65]. Moreover, signaling leading to activation of the mitogen-activated kinase (MAPK)/ERK pathway, such as growth factor receptor activation, or of transcription factors, such as serum response factor (SRF), early growth response gene 1 (EGR1), and also retinoic acid receptor (RAR) and PPAR, could participate in flotillin upregulation [66]. As these signaling pathways and transcription factors are often activated upon oncogenic stimulation, this could explain why flotillin upregulation occurs in so many different cancers. In addition, flotillin 1 expression is increased upon H-Ras expression in MCF10A mammary cells to increase tumor aggressiveness [7]. Moreover, in condition of suboptimal cancer treatment, metastasis formation is enhanced, as reported for small-size hepatocellular carcinoma with insufficient radiofrequency ablation. When hepatocellular carcinoma cells are heat-treated to mimic this process, flotillin upregulation is observed [67].

**Table 2:** MicroRNAs that regulate flotillin 1 and 2 gene expression during tumorigenesis

MicroRNA	Flotillin targeted	Cancer type	Source
miR-34a	*Flotillin 2*	Melanoma	[68]
miR-124	*Flotillin 1*	Kidney	[69]
miR-124	*Flotillin 1*	Breast	[16]
miR-133	*Flotillin 2*	Lung	[70]
miR-138	*Flotillin 1/2*	Esophagus	[71]
miR-182	*Flotillin 1*	Renal	[72]
miR-485	*Flotillin 2*	Lung	[73]
miR-485	*Flotillin 1*	Stomach	[63]
miR-449a	*Flotillin 2*	Stomach	[64]
miR-506	*Flotillin 1*	Kidney	[74]
miR-802	*Flotillin 2*	Prostate	[61]
miR-3908	*Flotillin 1*	Breast	[75]

Until now, no animal models mimicking flotillin upregulation were generated, except in *Drosophila*, where flotillin overexpression mutants showed perturbed adhesion molecule and morphogen distribution [76]. Moreover, flotillin upregulation in cellular models devoid of oncogenic pathway activation is sufficient to promote extracellular matrix (ECM) degradation, cell migration, and cell invasion [9]. This suggests that flotillin expression level is a crucial element in their function.

It is important to note that most of the studies on flotillins were carried out in tumor cell lines where flotillin expression levels are greatly increased. This implies that the majority of cellular functions attributed to flotillins since their discoveries were identified in a context of overexpression that favors their oligomerization, leading to plasma membrane remodeling and endocytosis. Moreover, at the molecular level, the functions of flotillins are usually associated with their local accumulation that is influenced by their expression level. This is the case for their role in cell signaling. Specifically, flotillins form membrane micro-domains where different receptors and proteins concentrate. As these regions act as signaling platforms, flotillins are associated with the regulation of different signaling processes. Similarly, most of the experiments that demonstrated flotillin function in endocytosis were performed in conditions of increased flotillin local concentration (after flotillin transfection and therefore overexpression). These experiments showed that the UFIT pathway is responsible for the endocytosis of several proteins towards flotillin-positive late endosomes, where their activity is modified and from where the cargos could be exocytosed and recycled at the cell surface in order to participate in tumorigenesis. Endocytic recycling and signaling are two intertwining processes of which flotillins emerge as key regulators.

### Flotillin-mediated endocytosis, a vesicular trafficking pathway exacerbated in cancer

As flotillins are present in the plasma membrane and in purified endosomes, Nichols’ group wanted to determine their role during endocytosis and showed that flotillins are found in endocytic vesicles, which are distinct from clathrin-coated pits and caveolae, and are involved in the internalization of protein cargos, such as the glycophosphatidylinositol (GPI)-anchor protein CD59 and the ganglioside GM1 [43, 77]. Moreover, pioneering studies revealed that overexpression of both flotillins induces flotillin hetero-oligomers that generate flotillin-positive plasma membrane micro-domains promoting plasma membrane curvature and the formation of endocytic vesicles [43]. Increasing the size of flotillin oligomers in HeLa cells, especially by incubation with epidermal growth factor, led also to their endocytosis from the plasma membrane [46]. Altogether, these studies carried out more than ten years ago suggested the involvement of flotillin micro-domains in endocytosis.

Since then, other protein cargos the UFIT pathway were identified, such as transmembrane and GPI-anchor proteins [9, 78–84] and extracellular proteins [85, 86] (Table 3). Most of them can enter into the cell through clathrin-mediated endocytosis or other clathrin-independent pathways, such as macro-pinocytosis. What orients the choice between flotillin-dependent or flotillin-independent endocytic pathways is not known, but the binding of a ligand could be an important parameter for transmembrane proteins. For example, the low-density lipoprotein receptor–related protein 6 (LRP6) is internalized by different endocytic routes, depending on ligand binding. In the presence of the ligand, LRP6 is endocytosed *via* a clathrin-dependent route that results in LRP6 trafficking to the lysosome for its degradation. Conversely, in the absence of ligand, LRP6 is endocytosed in a flotillin-dependent manner [84]. The choice of the flotillin-mediated endocytosis pathway for a given cargo could be explained by its presence in flotillin-rich membrane micro-domains, as shown using super-resolution microscopy for MT1-MMP [9], where the cargo can directly or indirectly interact with flotillins. Despite all these studies suggesting the existence of a flotillin-dependent endocytic pathway, its biological relevance and physiological relevance are challenged. Indeed, flotillin knockdown affects the clustering of several proteins at the plasma membrane, but their endocytosis is not totally impaired, probably because these protein cargos can also use other endocytic pathways. In addition, the absence of a marked phenotype in flotillin knockout mice that are viable and fertile [3, 4] does not argue in favor of an essential role of flotillins in endocytosis.

**Table 3:** Identified cargos of the UFIT pathway

Protein	Cell type, experimental context	Source
ALK	Neuroblastoma cells	[10]
APP	Neuroblastoma N2a cells	[83]
CD59 (GPI-anchor protein)	Polarized hepatocytes	[78]
Cholera toxin B	HeLa cells, human BeWo choriocarcinoma cells	[77, 87]
DAT (dopamine transporter)	Human embryonic kidney 293 (Hek293) cells	[79]
Leucine-Rich Amelogenin Peptide (LRAP)	Murine cementoblast cell line (OCCM-30)	[88]
LDL receptor-related protein 6 (LRP6)	HepG2 hepatocytes	[84]
MT1-MMP	MDA-MB-231 cells, MCF10A and C2C12 with flotillin upregulation	[9]
Muscarinic type 3 receptor (M3R)	Human submandibular gland (HSG) epithelial cells	[82]
PrPc	Human neuroblastoma SK-N-SH cell line, human embryonic kidney 293 (HEK293)	[89]
Niemann-Pick C1-like 1 (NPC1L1)	CRL-1601 rat hepatoma cell, cholesterol uptake	[81]
Proteoglycans	Hela cells, cationic polymers, lipids, and polypeptides uptake	[90]
Semaphorin 3A	Rat cortical neurons	[85]
Sticks-and-Stones (SNS), Roughest (Rst) and Kin-of-irre (Kirre)	*Drosophila* transmembrane proteins of the Ig superfamily protein. SNS binds heterophilically with Rst and Kirre	[76]

As previously mentioned, the involvement of flotillin in endocytosis initially emerged through approaches using artificial ectopic overexpression [43, 77]. As many publications showed that flotillin upregulation is a common feature of many invasive tumors, the relevance of flotillin-mediated endocytosis has regained interest recently. Unlike the moderate effect of flotillin loss of function, flotillin upregulation (gain of function) is harmful for tissue homeostasis because flotillin overexpression participates in tumor development and in *Drosophila* it disrupt intercellular adhesion, thus leading to embryonic lethality [76]. It will therefore be interesting to develop animal models in which flotillins can be upregulated in an inducible way to address *in vivo* the effect of their aberrant expression. One consequence of the UFIT pathway is the accumulation of flotillin-positive intracellular vesicles in the late endosomal compartment, particularly in endolysosomes [9, 57]. Currently, the role of flotillins in these intracellular vesicles is poorly described.

The molecular mechanisms of the UFIT pathway are not known. Local accumulation of flotillins at the plasma membrane is associated with the formation of invaginations [43]. Different proteins with properties similar to flotillins (i.e., membrane localization, oligomerization, actin binding) can bend membranes after their local accumulation [91]. Therefore, a local increase of flotillin concentration could be sufficient to induce membrane curvature and endocytosis. Experiments using optogenetics to force flotillin oligomerization to mimic this local concentration increase or *in vitro* approaches with artificial membranes in which flotillins are incorporated will allow validating such hypotheses. One can also imagine that flotillins can bend the membranes by recruiting different partners. This binding could be the direct results of the increased flotillin local concentration and/or of the generation of specific lipid domains. Indeed, flotillins affect sphingolipid distribution in membrane micro-domains. Specifically, flotillins bind to sphingosines through their SPFH region, and in the absence of flotillins, sphingosines in membrane micro-domains are impaired as well as the generation of sphingosine-1-phosphate [92]. Sphingosine-1-phosphate levels at the plasma membrane are associated with the formation of endocytic structures [93] and with the recruitment of Bin-Amphiphysin-Rvs (BAR) domain-containing endophilin A2 and B1 [94]. Interestingly, it was shown that endophilin A2 controls clathrin-independent endocytosis [95]. Other BAR domain–containing proteins, such as SNX, would be good candidate regulators of endocytosis promoted by flotillins because these proteins can bend the membranes and because flotillins are direct partners of SNX4 [96], a BAR domain protein that has already been implicated in endocytic mechanisms [97]. Some studies also suggested that flotillins participate in the recruitment of the clathrin-dependent endocytosis machinery. Indeed, amyloid precursor protein internalization requires the presence of flotillins and the recruitment of the AP2 adapter and clathrin, suggesting a mixed pathway [83]. Nevertheless, highly organized electron-dense clathrin-like coats were not detected at sites of plasma membrane invaginations in cells with upregulated flotillins. Similarly, the importance of dynamin in this process is unclear. Indeed, flotillin-mediated internalization of CTX-B [77] and Semaphorin 3A [85] but not of GPI-anchor proteins [78] seems to be independent of dynamin presence. Thus, depending on the cellular context and the cargos, different endocytic mechanisms of the UFIT pathway have been proposed. The identification of the mechanisms and machinery used to promote endocytic vesicle formation in the context of flotillin upregulation should be facilitated by the development of systems to force flotillin oligomerization without activation of additional signaling pathways.

### Flotillin role in the late endocytic compartment: the example of MT1-MMP

**Fig. 2:**
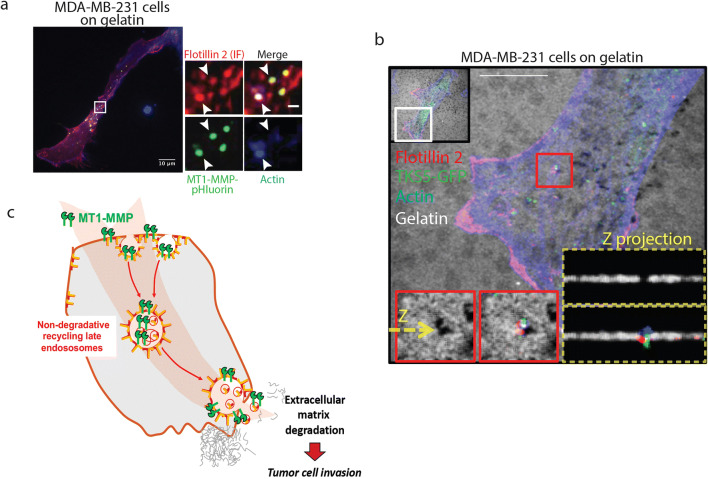
Flotillins and extracellular matrix degradation: delivery of flotillin-positive vesicles that contain MT1-MMP at degradation sites in breast cancer cells. **a** Confocal image of one MDA-MB-231 cell that expresses MT1-MMP-pHluorin (a protein that is fluorescent only at the extracellular pH of 7.4) cultured on non-fluorescent cross-linked gelatin and stained with an anti-flotillin 2 antibody (1:100, 610383, BD Biosciences) to identify the flotillin-positive vesicles and with Alexa Fluo 405 phalloidin (Thermo Scientific) to visualize F-actin. The zooms of the boxed region show that MT1-MMP is delivered to degradation sites by flotillin-positive vesicles. Confocal images were acquired using a Confocal Leica SP5-SMD microscope and a LEICA 63x/1.4 oil HCX PL APO CS objective controlled using the Leica LAS AF software. Bar, 10 μm. **b** Confocal image of one MDA-MB-231 cell that express flotillin 2-mCherry and TKS5-GFP cultured on Alexa Flour 633–conjugated fluorescent gelatin and stained with Alexa Fluo 405 phalloidin (Thermo Scientific) to visualize F-actin. The red boxed regions illustrate the matrix degradation site (as revealed by the degraded gelatin and the presence of TKS5 and actin) to which one flotillin-positive vesicle is delivered. This event is clearly seen in the higher magnification of the red boxed region and in the corresponding Z projections. Confocal images were acquired using a Confocal Leica SP5-SMD microscope and a LEICA 63x/1.4 oil HCX PL APO CS objective controlled using the Leica LAS AF software. Bar, 10 μm. **c** Model for the role of the UFIT pathway in MT1-MMP trafficking. The protein cargo MT1-MMP is present in flotillin-rich plasma membrane micro-domains and is endocytosed together with flotillins to reach flotillin-positive late endosomes that correspond to the endosomal MT1-MMP reservoir compartment. In this compartment, MT1-MMP is not degraded but exocytosed through flotillin-positive vesicles that are delivered to degradation sites

Upregulated (endogenous and ectopic) flotillins are preferentially found in intracellular vesicles defined as CD63-, RAB7-, and LAMP-1-positive late endosomes (Fig. 1d); however, their functions in this endocytic and recycling compartment are not very clear at the moment [9, 45, 51, 52, 54]. Late endosomes could maturate and fuse with lysosomes or be delivered to the cell periphery [98]. One key function of these non-degradative flotillin-positive late endosomes is to allow cargo recycling [9, 96]. This was clearly illustrated in the case of MT1-MMP, a membrane-tethered matrix metalloproteinase with a key role in the regulation of localized ECM breakdown [9]. Indeed, MT1-MMP is targeted to flotillin-rich late endosomes upon flotillin upregulation (Fig. 2a) to allow its recycling and delivery at degradation sites. When cells degrade the gelatin matrix, flotillin-positive vesicles are observed at degradation sites, called invadopodia and identified by TKS5 and F-actin, from which MT1-MMP is delivered to promote matrix degradation (Fig. 2b). Flotillin upregulation is associated with increased MT1-MMP exocytosis, leading to increased matrix degradation, a key process during tumor cell invasion (see model in Fig. 2c). Also, in a non-pathological context, flotillins are involved in invasion and especially in ECM degradation. Indeed, flotillin inhibition in macrophages induces a decrease in gelatin matrix degradation in 2D. In this model, through their interaction with the kinesin KIF9, flotillins appear to regulate the formation of ECM degradation structures [99]. Interestingly, kinesins are involved in MT1-MMP transport in tumor cells [100]. We can hypothesize that flotillin micro-domains serve as a membrane platform for KIF9 recruitment at vesicles, thus promoting the transport of metalloproteases. However, this possible function has not been thoroughly investigated yet.

In biochemical assays, flotillin 2, through its SPFH domain, binds to proteins involved in recycling pathways, such as Rab11 and SNX4; however, flotillin co-localization with these proteins has not often clearly observed in various cell types, even cancer cells with upregulated flotillins (see Fig. 1d for MDA-MB-231 cells) [9, 96]. How the UFIT pathway influences the activity and/or recruitment of these proteins along the endocytic pathway remains to be determined. Flotillins also bind to Hrs, a protein of the ESCRT-0 complex, and could affect Hrs-mediated cargo sorting to control their degradation [101]. Moreover, the ESCRT complex also has a role in protein trafficking from the late endosomal compartment to the plasma membrane [102]. Therefore, more studies on the role of flotillins in these late endosomes are needed to clarify their function in this compartment where they accumulate when upregulated, such as in cancer cells.

### Flotillins and signaling

Several independent studies reported that flotillins influence the activation of signaling pathways that promote EMT, cellular adhesion perturbations, and cellular invasion. In these last years, the number of publications that identified a role for flotillins in the regulation of oncogenic signaling pathways has increased (Table 4). Many of these studies were performed by decreasing flotillin expression levels in tumor cells and demonstrated that flotillins are necessary for the activation of oncogenic signaling pathways. However, studying the consequences of flotillin upregulation, which is accompanied by a gain-of-function effect, is required particularly to determine whether their overexpression is sufficient to induce downstream effects. Indeed, recently, ectopic overexpression of flotillins is used again to analyze their functions. For example, in cellular models of lung adenocarcinoma, overexpression of flotillins is sufficient to strongly induce cell invasion [103]. Similarly, in models of hepatocellular carcinoma, overexpression of flotillins is sufficient to induce a Raf/MEK/ERK1/2-dependent signaling cascade. This leads to EMT activation and increased cell invasion *in vitro* and to metastasis formation *in vivo* [11]. Finally, in a model of nasopharyngeal carcinoma, overexpression of flotillins induces EMT and consequently increases cell migration and invasion [5, 29]. The molecular mechanism seems to depend on the secretion of TGF-β1 and activation of the TGF-β/SMAD3, PI3K/Akt3, and NF-κB signaling pathways. These examples are not exhaustive and every year new studies show that overexpression of flotillins is sufficient to acquire invasive properties in different cancer cell types (Table 4). All these studies that identified a key role of flotillins in signaling pathways involved in cancer development were performed using cancer cell lines with many mutations in genes with roles in tumor induction and invasion. Therefore, to specifically identify the precise contribution of flotillin upregulation in the activation of these key signaling pathways during tumor development, cellular models devoid of these mutations should be used in the future. Upregulation of flotillins in normal cells is sufficient to acquire invasive properties and promote their capacity to degrade the ECM [9]. It is important to emphasize that overexpressed flotillins can simultaneously activate several signaling pathways. Therefore, preventing/limiting flotillin upregulation could have a far superior therapeutic efficacy compared with approaches in which one single protein kinase or signaling pathway is inhibited.

**Table 4:** Flotillins and activation of oncogenic signaling pathways

Model used	Flotillin	Pathway affected	Source
Transgenic mouse model (MMTV-PyMTxFlot2^−/−^)	Flotillin 2 KO	No impact on primary tumor formation, reduced lung metastases	[12]
Breast cells	Flotillin 1 KD	Decreased H-Ras, Rac1, p38 and PI3K/AKT activation	[7]
	Flotillin 1 and 2 KD	Decreased ERBB2 phosphorylation and AKT activation	[14]
	Flotillin 1 and 2 KD	Decreased cell proliferation, AKT activation and cyclin D1 expression, increased p21 and p27 expression	[104]
	Flotillin 1 and 2 KD	Decreased cell proliferation, cell migration and invasion	[16]
	Flotillin 1 and 2 KD	Decreased cell matrix degradation and invasion	[9]
	Flotillin 1 KD	Increased EGFR and ERK/MAPK activation (restricted to the MCF7 cell line)	[105]
Colorectal cells	Flotillin 1 and 2 KD	Decreased resistance to doxorubicin, increased apoptosis	[106]
Gastric cells	Flotillin 2 KD	Decreased ERBB2 level	[22]
Glioma cells	Flotillin 2 KD	Decreased cell viability, migration, and invasion	[35]
Hepatocellular carcinoma cells	Flotillin 2 upregulation and KD	Increased flotillin levels promote proliferation, EMT, invasion, and tumor growth (xenografts in mice)	[11]
Melanoma cells	Flotillin 2 upregulation	Increased proliferation and metastases formation (xenografts in mice), increased PAR-1 expression	[6]
Nasopharyngeal cells	Flotillin 2 upregulation and KD	Increased flotillin levels promote NF-kB and PI3K/AKT3 activation	[8]
	Flotillin 2 KD	Decreased TGFβ-induced EMT	[29]
	Flotillin 1 upregulation and KD	Increased flotillin levels promote metastases to lymph node and activation of the TGF-β pathway	[5]
Neuroblastoma cells	Flotillin 1 and 2 KD	Flotillin 1 control ALK distribution and activity	[10]
Prostate cancer cells	Flotillin 1	Sumoylation of non-palmitoylated flotillin 1 promotes its nuclear localization and stabilizes Snail	[56]
Skin carcinoma	Flotillin 2 KD	Inhibition of EGFR internalization and perturbation of E-cadherin-mediated cell-cell adhesion	[107]

The molecular mechanisms of activation of oncogenic pathways by the UFIT pathway remain poorly identified. Flotillins could have key functions at different cellular levels. First, flotillins at the plasma membrane participate in the clustering of membrane proteins, such as the receptor tyrosine kinases ErbB2 and EGFR involved in the activation of oncogenic signaling pathways [14, 108]. In neuroblastoma, flotillin 1 was identified from a screen as a phosphotyrosine-containing protein associated with the oncogenic anaplastic lymphoma kinase (ALK) and was shown to control ALK activity through its stabilization at the plasma membrane [10]. Moreover, when flotillins are upregulated, like in tumor cells, they accumulate in non-degradative late endosomes with cargos of the UFIT pathway such as MT1-MMP [9]. It is now important to determine the role of flotillins in late endosomes, particularly their contribution to the maintenance of the signaling pathways starting at the plasma membrane. Endocytosis contributes to downregulate incoming signals, but in some cases signaling pathways could be maintained in early and late endosomes [109]. Whether and how flotillins participate in the decision to degrade or not the endocytosed material remains to be determined. Interestingly, flotillin 1 directly interacts with Raf, MEK1/2, and ERK1/2 [108], but it is not known whether flotillins could regulate their recruitment to signaling endosomes.

Finally, post-translational modifications, such as phosphorylation and sumoylation, also could modify flotillin functions. For instance, it has been proposed that sumoylation of a non-palmitoylated form of flotillin 1 favors Snail stabilization and Snail-mediated EMT gene expression in prostate cancer cells [56].

In conclusion, flotillins emerged these last years as proteins that play a pivotal role in a broad spectrum of human cancers. For the future structure and function analysis of the flotillin-containing membrane domains at the nanoscale level in living cells, the use of high-resolution microscopy techniques, such as STED, SIM, PALM, and dSTORM, will be very useful. Combining these approaches with new lipid dyes will allow the precise identification of the properties of membrane micro-domains. Above all, the development of new tools and animal models to study flotillin upregulation in a temporal and spatial controlled manner is required to better understand their mechanism of action and their role in pathologies, such as cancer. Particularly, identifying the role of flotillins in the late endosomal recycling compartment and how the UFIT pathway influences the dynamic turnover and fate of different protein cargos will be an important step.
